# Medical marijuana knowledge and attitudes amongst internal medicine residents

**DOI:** 10.1186/s12875-022-01651-9

**Published:** 2022-03-03

**Authors:** Iman Makki, Binbin Zheng-Lin, Maanit Kohli

**Affiliations:** 1 Department of Medicine, Mount Sinai Morningside and Mount Sinai West, Icahn School of Medicine at Mount Sinai, New York, NY USA; 2 Division of Hospital Medicine, Department of Medicine, Mount Sinai Morningside and Mount Sinai West, Icahn School of Medicine at Mount Sinai, 1000 10th Ave, New York, NY 10019 USA

**Keywords:** Medical marijuana, Internal Medicine residency, Postgraduate training, Chronic pain, Substance abuse, Medical education

## Abstract

**Background:**

Mounting evidence suggests the safety and efficacy of medical marijuana (MM) in treating chronic ailments, including chronic pain, epilepsy, and anorexia. Despite incremental use of medical and recreational cannabinoids, current limited evidence shows generalized unpreparedness of medical providers to discuss or recommend these substances to their patients. Herein, the present study aims to examine internal medicine residents’ knowledge of marijuana and their attitude towards its medical use.

**Methods:**

This is a descriptive cross-sectional study. A survey with 12 standardized queries was created and distributed among the internal medicine residents from Mount Sinai Morningside-West (MSMW) program from July 2020 to December 2020. Participants included preliminary and categorical residents from post-graduate years one to three. The survey consisted of self-assessment of residents’ knowledge on the indication, contraindication, adverse effects of MM.

**Results:**

Eighty-six (59%) out of 145 residents completed the questionnaire. Despite most trainees (70%) having considered certifying the use of MM for their patients, over 90% reported none to little knowledge on its use. Approximately 80% of the surveyed residents expressed willingness to receive an appropriate educational curriculum.

**Conclusion:**

To the best of our knowledge, this is the first study that indicated a critical lack of medical marijuana-related knowledge in surveyed internal medicine residents. In a population with growing cannabis consumption, physician training on the indication, toxicity, and drug interaction of cannabinoids is warranted.

## Introduction

Mounting studies suggest the therapeutic efficacy of cannabis and its derivatives, tetrahydrocannabinol (THC) and cannabidiol (CBD), in debilitating conditions such as chemotherapy-induced nausea, anorexia, epilepsy, multiple sclerosis, and amyotrophic lateral sclerosis [1–3]. Moreover, amidst the opioid epidemic, the use of MM may offer a new therapeutic pathway for chronic noncancerous and cancer-related pain. Despite incremental use of medical and non-medical cannabinoids, physicians have reported gaps in their knowledge related to these substances [4]. In this study, we examine the knowledge of internal medicine (IM) residents on marijuana and their attitude towards its medical use.

## Methods

In this descriptive cross-sectional study, IM residents from Mount Sinai Morningside-West (MSMW) program were surveyed from July 2020 to December 2020. The data was collected for a medical education-based needs assessment survey. All trainees from the post-graduate year (PGY) one to three were eligible and included preliminary and categorical residents. An anonymous online questionnaire with 12 standardized queries was created using SurveyMonkey® and distributed via Microsoft® Teams, institutional emails, and smartphone messaging applications. The survey consisted of demographics questions, as well as five-point scale questions about trainees’ self-assessment on MM use, and their stance on MM education during residency training. The full questionnaire is available in Table 1.

**Table 1:** Full survey with twelve standardized questions

Choose from 1- no knowledge, 2- very little knowledge, 3- some knowledge, 4- substantial knowledge, and 5- high level of knowledge:	
Q1	Would You consider Prescribing Medical Marijuana for your patient?
Q2	How would you describe your knowledge about medical marijuana?
Q3	How would you describe your knowledge about Indications or Uses of Medical Marijuana?
Q4	How would you describe your knowledge about Risks / Side Effects and Contraindications of Medical Marijuana?
Q5	How would you describe your knowledge about marijuana dosage?
Q6	How would you describe your knowledge about the different types/forms of marijuana products?
Choose from 1- disagree, 2- somewhat disagree, 3- neither agree nor disagree, 4- somewhat agree, and 5- agreed:	
Q7	Do you feel you have adequate knowledge of where to find information about medical marijuana?
Q8	Is there a need for more medical education about Marijuana?
Demographic questions:	
Q9	What is your gender?
Q10	What is your age?
Q11	Which race/ethnicity best describes you?
Q12	What is your post-graduate training year?

MSMW IM residency is an ACGME-accredited program affiliated to Mount Sinai Icahn School of Medicine (New York City, New York). This is one of the largest academic programs in the United States and is composed of a preliminary internship of 20 trainees and a categorical residency with 125 residents. Approximately 74% of the in-training physicians are international medical graduates.

## Results

Eighty-six (59%) out of 145 residents completed the questionnaire, of which 39 (45%) and 47 (55%) were female and male, respectively. 80 (93%) were aged 25 to 34 years. Of participants who chose to disclose their ethnicity, 29 (34%) self-identified as Caucasian, whereas 15 (17%) were Hispanic. PGY-1 trainees had the highest participation rate of 42%, followed by 32% in PGY-2and 26% in PGY-3.

**Fig. 1:**
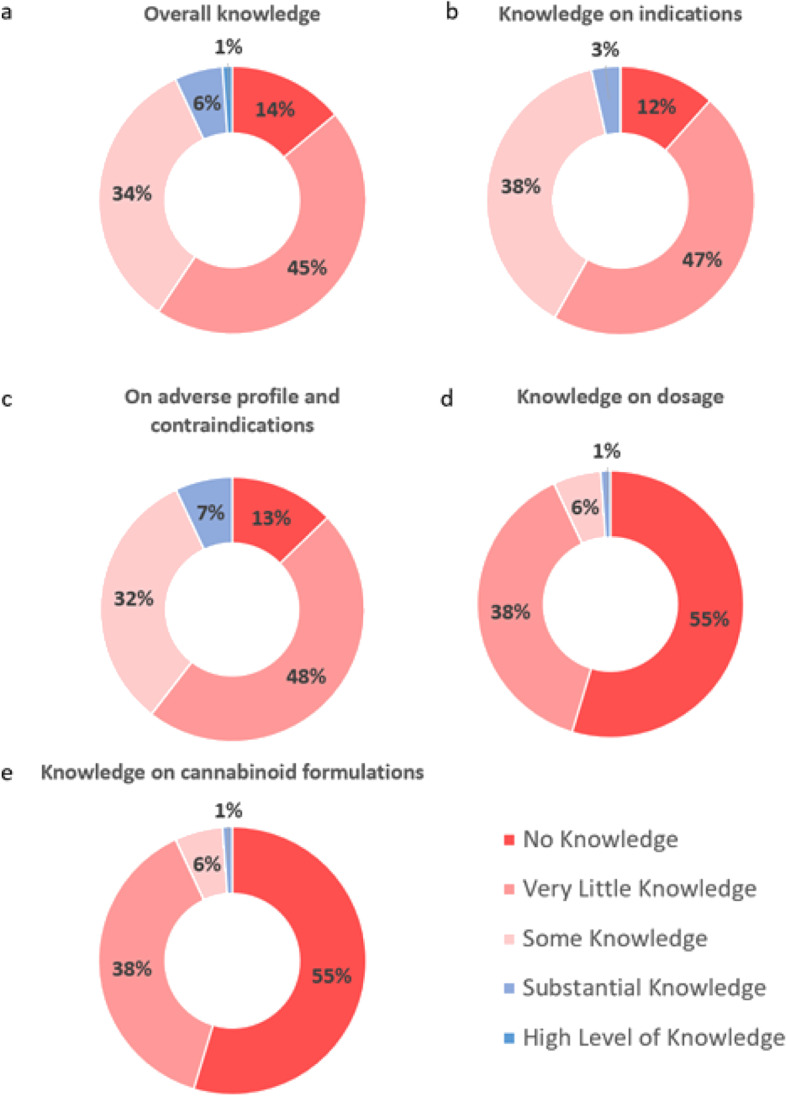
In-training Internal Medicine Residents Self-Perception of Their Knowledge on Medical Cannabis

**Fig. 2:**
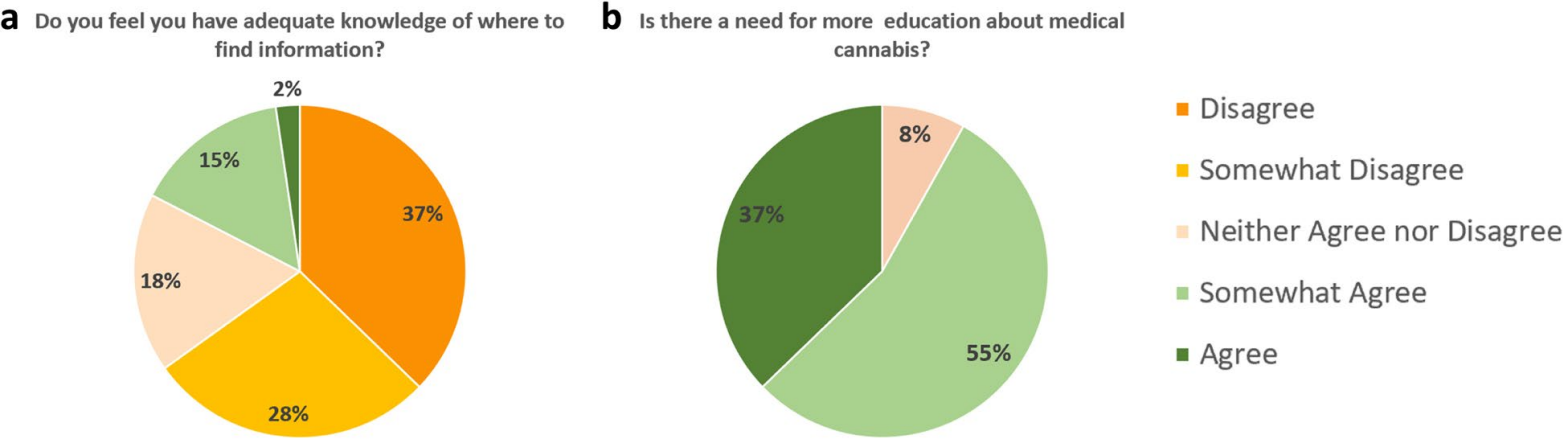
Internal Medicine Residents’ Opinion on Resources and Education on Medical Cannabis

While the majority (*n*=60, 70%) of trainees had considered certifying medical cannabinoids for their patients, only six (7%) residents felt they had an adequate amount of knowledge (Fig. 1). Regarding indications and contraindications of medical marijuana, almost all residents endorsed having none to some knowledge (respectively *n*=83 [97%] and *n*=80 [93%]). Eighty-five (99%) expressed none to some familiarity with marijuana dosing, whereas only four (5%) reported substantial knowledge on different formulations of medical cannabinoid products. Of notable significance, 71 (83%) participants were unsure where to find pertinent information, and 79 (92%) agreed on the necessity of implementing medical education on this substance (Fig. 2).

## Discussion

Chronic pain affects 20% of the general population and over 50% of cancer patients. Uncontrolled chronic pain impairs quality of life, exacerbates pre-existing comorbidities, and may lead to depression and anxiety [5, 6]. Opioids remain one of the mainstay treatments for chronic cancer-related pain. Developing opioid tolerance due to chronic use, significant adverse effects, and potential for addiction are deterrent factors that may lead to pain undertreatment in cancer patients [7, 8]. Furthermore, patients with severe chronic pain are at risk of accidental opioid overdose, especially when used concomitantly with benzodiazepines and/or alcohol [9].

Accumulating evidence suggests the safety and efficacy of MM in the treatment of both chronic non-cancerous pain and cancer-related pain. A systematic review with thirty-six trials assessed the efficacy of different MM formulations (sprays, oral, and smoked) to treat non-cancer neuropathic and non-neuropathic pain. In the sixteen evaluable trials, prolonged cannabinoid treatment (two to eight weeks), as opposed to placebo, was associated with a significantly greater reduction in pain (weighted mean difference on a pain visual analog scale was −0.68, 95% confidence interval −0.96 to −0.40, I2 = 8%, *P* <0.00001) [1]. In patients with advanced cancer with moderate chronic pain, Lichtman et al. observed that administration of nabiximols spray was associated with a median 15.5% improvement in average pain score as opposed to 6.3% with placebo (*P*=0.0378) [3]. In an observational study, different formulations of THC and CBD (oil, capsules, and cigarettes) were provided to 3,619 patients with severe cancer-related pain (median baseline pain score of 8/10) [2]. In participants who remained on cannabis at six-month follow-up (*N*=1997, 55%), only 4.6% reported ongoing severe pain. Concomitant opioid use was decreased and stopped respectively in 10 and 36% of patients.

MM showed a positive safety profile in these studies. Treatment-related adverse events were reportedly mild to moderate, and the most common effects were somnolence, dry mouth, increased appetite, dizziness [1–3, 10]. Of significant note, fatal overdose secondary to marijuana use is uncommon as opposed to opioids which caused more than 46,000 deaths per year [11]. However, the majority of patients diagnosed with E-cigarette or vaping product use associated lung injury (EVALI) have reported use of THC-containing smokeless products [12, 13] and, albeit rare, deaths have been reported [14]. Moreover, there is lacking evidence on long-term effects of cannabinoids.

The United States is experiencing progressive legalization of marijuana for recreational and medicinal purposes. Notwithstanding, limited evidence points to low levels of education on medical cannabis amongst healthcare professionals. In a survey distributed to pharmacy students from six colleges in Ohio, students endorsed low medical marijuana knowledge and felt more education is needed in pharmacy schools [15]. In the same study, medical marijuana education was provided in less than two-thirds of 79 colleges located in MM legal states. In another single-center Spanish study, surveyed nursing students were unaware of indications of medical marijuana. Less than one percent received some type of education regarding cannabis, while more than 85% felt the need to incorporate cannabis education to their curriculum [16]. In another study by Philpot et al., 62 primary healthcare providers in a large healthcare system in Minnesota were surveyed about their attitudes and beliefs about MM. Similar to our findings, their survey concluded that a significant proportion of providers were not comfortable answering patients’ questions about MM, though were willing to receive more related education [4].

To the best of our knowledge, this is the first study to observe a critical lack of knowledge in MM in in-training IM residents. Hence, it is worth implementing a curriculum for resident physicians that includes indications, medication interactions, and side effects of MM use. Questions related to substance use disorder comprised less than two percent in the American Board of Internal Medicine (ABIM) certification exam [17]. Medical Knowledge Self-Assessment Program (MKSAP) offered by the American College of Physicians (ACP), a popular study material for ABIM certification exam used by internal medicine residents, does not include any detailed section regarding the indications, contraindications, or adverse effects of cannabinoids [18]. This might lead to a lack of preparedness of residents as frontline providers. Indeed, cannabis use is augmenting due to the increasing number of legalizing states, expansion of its off-label uses, and generalization of recreational uses. Physicians should be familiarized with its use, as well as with the management of MM-related toxicities. At the same time, qualifying conditions for MM use vary depending on the state of practice. Local graduate medical education offices should incorporate pertinent curriculum while keeping up pace with federal and state policy changes.

Our survey results were based on a single-institution experience from an academic center located in New York. A significant percentage of surveyed trainees obtained their medical degrees from outside countries. Nevertheless, the present study emphasizes the lack of formal education on medical cannabis during IM residency training and not before it. Albeit the survey participation was limited, we believe the results were relevant as they show a general trend towards a lack of knowledge on medical cannabis and point to the necessity of a formal educational curriculum.

## Limitations

The study is a descriptive cross-sectional study at a single institution and may not be extendible to residencies or IM programs across the country. Additionally, our study seeks the residents to self-rate their knowledge and attitude regarding MM use.

## Conclusion

The consumption of medical and nonmedical cannabis is growing. Our study highlighted a needs assessment for MM education at our institution, with the results recognizing the resident's self-awareness about their lack of knowledge. Frontline providers like in-training residents should be familiarized with MM. Analogous studies, if done in other programs across the country, may yield similar results resulting in a more extensive discussion about the need for curriculum development.

### Ethical declarations

Our study “Medical Marijuana Knowledge and Attitudes amongst Internal Medicine Residents” did not consist of any experimental protocols. In this descriptive cross-sectional study, IM residents from Mount Sinai Morningside-West (MSMW) program voluntarily responded to the distributed survey. All methods were carried out in accordance with relevant guidelines and regulations. The IRB determined that the proposed activity is not research involving human subjects as defined by DHHS and FDA regulations. The data set presented is the secondary use of information collected for purposes other than research (medical education-based needs assessment). The holder of any identifiable data would never share those identifiers, nor could data be linked to any identifiable information. For that reason, the Federal Regulations did not apply, including obtaining consent. Accordingly, the IRB determined this project to be Not Human Subjects (NHSR) research.

## Data Availability

The datasets used and/or analyzed during the current study are available from the corresponding author on reasonable request.

## Abbreviations

ABIM: American Board of Internal Medicine
ACP: American College of Physicians
CBD: Cannabidiol
FDA: Food and Drug Administration
IM: Internal medicine
MKSAP: Medical Knowledge Self-Assessment Program
MM: Medical marijuana
MSMW: Mount Sinai Morningside-West
PGY: Post-graduate year
THC: Tetrahydrocannabinol
